# Redetermination of 6,6′-dimeth­oxy-2,2′-[hexane-1,6-diylbis(nitrilo­dimethyl­idyne)]diphenol

**DOI:** 10.1107/S1600536811020599

**Published:** 2011-06-04

**Authors:** M. Tabatabaee, M. R. Fotuhiardakani, Alan J. Lough

**Affiliations:** a Department of Chemistry, Yazd Branch, Islamic Azad University, Yazd, Iran; b Department of Chemistry, University of Toronto, Toronto, Ontario, Canada M5S 3H6

## Abstract

The title compound, C_22_H_28_N_2_O_4_, contains two independent centrosymmetric mol­ecules (*A* and *B*). In the previous structure determination [Xia *et al.* (2007 ▶). *Acta Cryst.* E**63**, o259] both *A* and *B* were modelled as neutral mol­ecules with the H atoms of the the O—H groups included in calculated positions. In this redetermination, the transferrable H atoms were located in difference maps and freely refined, indicating that one mol­ecule (*A*) crystallizes in the neutral (nonzwitterionic) form and the other in the zwitterionic form, namely 6,6′-dimeth­oxy-2,2′-[hexane-1,6-diylbis(nitrilo­dimethyl­idyne)]­di­phenol–6,6′-dimeth­oxy-2,2′-[hexane-1,6-diylbis(nitrilio­di­methyl­idyne)]diphenolate (1/1). This finding is supported by significant differences in the C—O(H) (*A*) and C—O^−^ (*B*) bond lengths. In the crystal, the zwitterionic mol­ecules (*B*) are involved in inter­molecular N—H⋯O hydrogen bonds forming one-dimensional chains along [001]. Each independent mol­ecule forms an intra­molecular O—H⋯N (*A*) or N—H⋯O (*B*) hydrogen bond. In mol­ecule *B*, one of the –CH_2_– groups is disordered over two sets of sites with refined occupancies of 0.659 (8) and 0.341 (8).

## Related literature

For background to Schiff bases as ligands, see: Ray *et al.* (2008 ▶); Tabatabaee *et al.* (2006 ▶). For the previous crystal structure of the title compound, see: Xia *et al.* (2007 ▶).
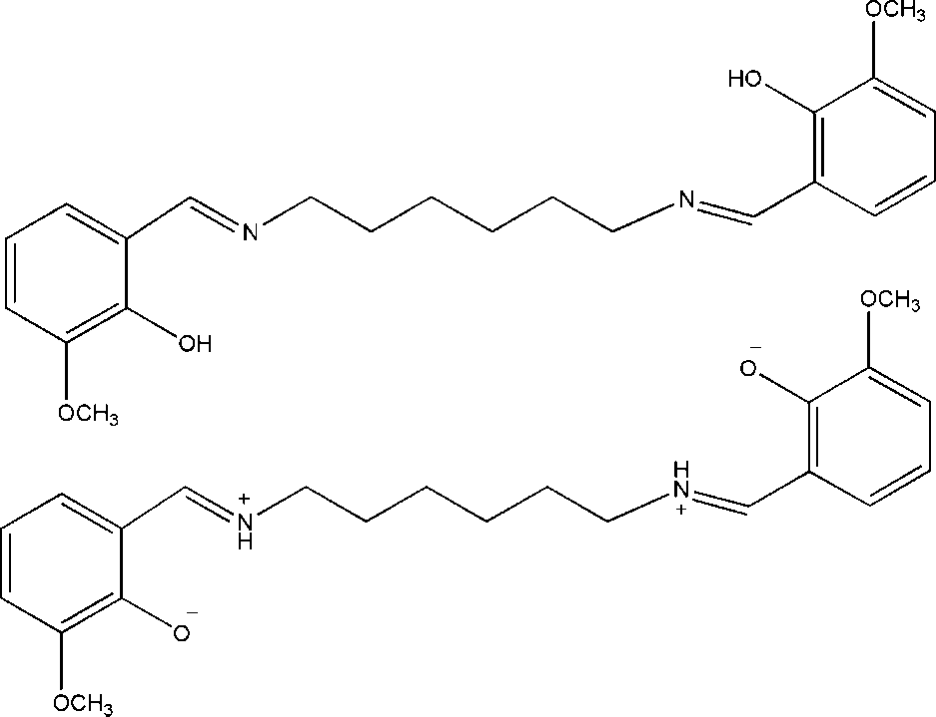


## Experimental

### 

#### Crystal data

C_22_H_28_N_2_O_4_
*M* *_r_* = 384.46
Monoclinic, 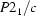

*a* = 21.2660 (4) Å
*b* = 8.4296 (3) Å
*c* = 11.1215 (9) Å
β = 92.3440 (17)°
*V* = 1992.02 (18) Å^3^
*Z* = 4
Mo *K*α radiation
μ = 0.09 mm^−1^
*T* = 150 K
0.32 × 0.24 × 0.18 mm

#### Data collection

Nonius KappaCCD diffractometer
Absorption correction: multi-scan (*SORTAV*; Blessing, 1995 ▶) *T*_min_ = 0.871, *T*_max_ = 0.990
9462 measured reflections
3462 independent reflections
1976 reflections with *I* > 2σ(*I*)
*R*_int_ = 0.042

#### Refinement

*R*[*F* ^2^ > 2σ(*F* ^2^)] = 0.075
*wR*(*F* ^2^) = 0.247
*S* = 1.05
3462 reflections
268 parameters
6 restraints
H atoms treated by a mixture of independent and constrained refinement
Δρ_max_ = 0.35 e Å^−3^
Δρ_min_ = −0.39 e Å^−3^

### 

Data collection: *COLLECT* (Nonius, 2002 ▶); cell refinement: *DENZO–SMN* (Otwinowski & Minor, 1997 ▶); data reduction: *DENZO–SMN*; program(s) used to solve structure: *SIR92* (Altomare *et al.*, 1994 ▶); program(s) used to refine structure: *SHELXTL* (Sheldrick, 2008 ▶); molecular graphics: *PLATON* (Spek, 2009 ▶); software used to prepare material for publication: *SHELXTL*.

## Supplementary Material

Crystal structure: contains datablock(s) global, I. DOI: 10.1107/S1600536811020599/hb5887sup1.cif

Structure factors: contains datablock(s) I. DOI: 10.1107/S1600536811020599/hb5887Isup2.hkl

Additional supplementary materials:  crystallographic information; 3D view; checkCIF report

## Figures and Tables

**Table 1:** Hydrogen-bond geometry (Å, °)

*D*—H⋯*A*	*D*—H	H⋯*A*	*D*⋯*A*	*D*—H⋯*A*
O1*A*—H1O⋯N1*A*	1.05 (5)	1.64 (5)	2.575 (4)	146 (4)
N1*B*—H2O⋯O1*B*	0.98 (5)	1.87 (5)	2.655 (4)	136 (4)
N1*B*—H2O⋯O1*B*^i^	0.98 (5)	2.31 (5)	2.976 (4)	125 (4)

Symmetry code: (i) 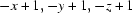.

**Fig. 1.**
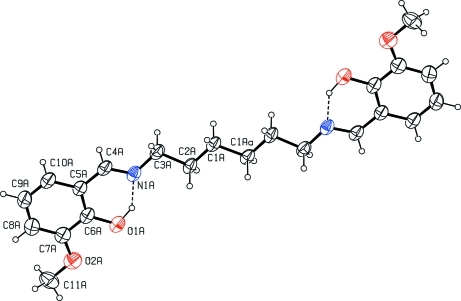
Molecule A showing 30% probability ellipsoids. An intramolecular hydrogen bond is shown with a dashed line. Symmetry code: (a) -x, -y + 2, -z + 1.

**Fig. 2.**
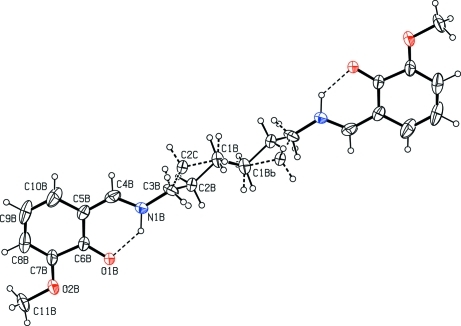
Molecule B showing 30% probability ellipsoids. An intramolecular hydrogen bond and the disorder is shown with a dashed lines. Symmetry code: (b) -x + 1, -y + 1, -z.

**Fig. 3.**
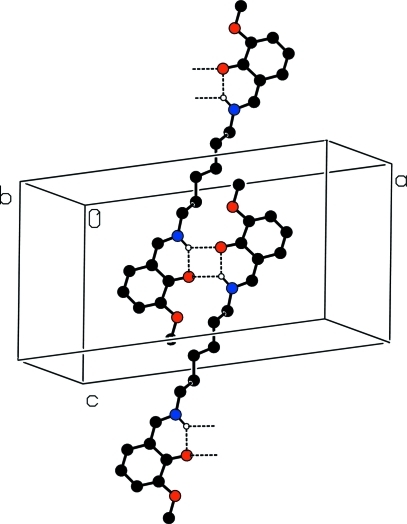
Part of the crystal structure with intermolecular hydrogen bonds shown as dashed lines. Only molecule B is shown but the disorder is not shown.

## supplementary crystallographic information

### Comment

Schiff base ligands of salicylaldehyde and diamine can act as tetradentate ligands and provide suitable coordination modes for transition metal ions (Ray *et al.* 2008). As part of our studies on Schiff bases and their complexes (Tabatabaee *et al.*, 2006) we have re-determined the crystal structure of the title compound, (I).

The title compound contains two centrosymmetric independent molecules [A and B] (see Figs. 1 and 2). In the original crystal structure determination (Xia *et al.*, 2007) the H atoms of the the N—H groups were included in calculated positions. In the current determination we refined the positional and isotropic displacement parameters of these H atoms which shows that one independent molecule [B], crystallizes in the zwitterionic form. This finding is supported by the significant differnces in the distances of the C6A—O1A and C6B—O1B bonds. The zwitterionic molecules [B] are involved in intermolecular N—H···O hydrogen bonds forming one-dimensional chains along [001] (see Fig. 3). Each independent molecule forms an intramolecular O—H···N (A) or N—H···O (B) hydrogen bond. In molecule B one of the –CH_2_– groups is disordeered over two sets of sites (Fig. 2) with refined occupancies 0.659 (8) and 0.341 (8). In one of the independent molecules in the original determination (Xia *et al.*, 2007) the anisotropic displacement ellipsoids of the C atoms in the hexyl chain are significantly larger than in the other.

### Experimental

All purchased chemicals were of reagent grade and used without further purification. A solution of hexamethylenediamine (1.162 g, 10 mmol) in EtOH (30 ml) was treated with 2-hydroxy-3-methoxybezaldehyde (3.043 g, 20 mmol) and the resulting mixture was acidified with 37% hydrochloric acid (10 drops). The reaction mixture was refluxed for 6 h. The progress of the reaction was monitored by TLC using hexane/ethylacetate (1/2) as eluent. After completion of reaction, the solid residue was filtered and washed with cold ethanol (10 ml). The filtrate was dissolved in CH_3_OH and kept at 277 K. Orange blocks of (I) were obtained after a few days (yield 82%).

### Refinement

Hydrogen atoms bonded to C atoms were placed in calculated positions with C—H distances ranging from 0.95 to 0.99 Å and included in the refinement in a riding-model approximation with *U*_iso_(H) = 1.2*U*_eq_(C) or *U*_iso_(H) = 1.5*U*_eq_(C) for methyl H atoms. H atoms bonded to O and N atoms were located in difference maps and refined independently with isotropic displacement parameters.

### Crystal data

**Table:**

C_22_H_28_N_2_O_4_	*F*(000) = 824
*M**_r_* = 384.46	*D*_x_ = 1.282 Mg m^−^^3^
Monoclinic, *P*2_1_/*c*	Mo *K*α radiation, λ = 0.71073 Å
Hall symbol: -P 2ybc	Cell parameters from 8545 reflections
*a* = 21.2660 (4) Å	θ = 2.6–27.5°
*b* = 8.4296 (3) Å	µ = 0.09 mm^−^^1^
*c* = 11.1215 (9) Å	*T* = 150 K
β = 92.3440 (17)°	Block, orange
*V* = 1992.02 (18) Å^3^	0.32 × 0.24 × 0.18 mm
*Z* = 4	

### Data collection

**Table:**

Nonius KappaCCD diffractometer	3462 independent reflections
Radiation source: fine-focus sealed tube	1976 reflections with *I* > 2σ(*I*)
graphite	*R*_int_ = 0.042
Detector resolution: 9 pixels mm^-1^	θ_max_ = 25.0°, θ_min_ = 2.6°
φ scans and ω scans with κ offsets	*h* = −20→25
Absorption correction: multi-scan (*SORTAV*; Blessing, 1995)	*k* = −9→9
*T*_min_ = 0.871, *T*_max_ = 0.990	*l* = −13→13
9462 measured reflections	

### Refinement

**Table:**

Refinement on *F*^2^	Primary atom site location: structure-invariant direct methods
Least-squares matrix: full	Secondary atom site location: difference Fourier map
*R*[*F*^2^ > 2σ(*F*^2^)] = 0.075	Hydrogen site location: inferred from neighbouring sites
*wR*(*F*^2^) = 0.247	H atoms treated by a mixture of independent and constrained refinement
*S* = 1.05	*w* = 1/[σ^2^(*F*_o_^2^) + (0.1195*P*)^2^ + 1.1215*P*] where *P* = (*F*_o_^2^ + 2*F*_c_^2^)/3
3462 reflections	(Δ/σ)_max_ < 0.001
268 parameters	Δρ_max_ = 0.35 e Å^−^^3^
6 restraints	Δρ_min_ = −0.39 e Å^−^^3^

### Special details

**Table:**

Geometry. All e.s.d.'s (except the e.s.d. in the dihedral angle between two l.s. planes) are estimated using the full covariance matrix. The cell e.s.d.'s are taken into account individually in the estimation of e.s.d.'s in distances, angles and torsion angles; correlations between e.s.d.'s in cell parameters are only used when they are defined by crystal symmetry. An approximate (isotropic) treatment of cell e.s.d.'s is used for estimating e.s.d.'s involving l.s. planes.
Refinement. Refinement of *F*^2^ against ALL reflections. The weighted *R*-factor *wR* and goodness of fit *S* are based on *F*^2^, conventional *R*-factors *R* are based on *F*, with *F* set to zero for negative *F*^2^. The threshold expression of *F*^2^ > σ(*F*^2^) is used only for calculating *R*-factors(gt) *etc*. and is not relevant to the choice of reflections for refinement. *R*-factors based on *F*^2^ are statistically about twice as large as those based on *F*, and *R*- factors based on ALL data will be even larger.

### Fractional atomic coordinates and isotropic or equivalent isotropic displacement parameters (Å^2^)

**Table:**

	*x*	*y*	*z*	*U*_iso_*/*U*_eq_	Occ. (<1)
O1A	0.16093 (12)	0.6165 (3)	0.8801 (3)	0.0706 (8)	
O2A	0.22393 (14)	0.4763 (4)	1.0569 (3)	0.0894 (10)	
N1A	0.08999 (14)	0.5965 (3)	0.6874 (3)	0.0660 (9)	
C1A	0.00468 (18)	0.9101 (4)	0.4966 (3)	0.0648 (10)	
H1A1	−0.0371	0.8580	0.4966	0.078*	
H1A2	0.0238	0.8842	0.4193	0.078*	
C2A	0.04555 (17)	0.8419 (4)	0.5979 (3)	0.0655 (11)	
H2AA	0.0261	0.8645	0.6754	0.079*	
H2AB	0.0872	0.8943	0.5991	0.079*	
C3A	0.0543 (2)	0.6646 (4)	0.5851 (4)	0.0741 (11)	
H3AA	0.0125	0.6131	0.5778	0.089*	
H3AB	0.0765	0.6426	0.5105	0.089*	
C4A	0.08887 (18)	0.4456 (4)	0.7018 (3)	0.0650 (11)	
H4AA	0.0648	0.3830	0.6458	0.078*	
C5A	0.12288 (17)	0.3671 (4)	0.8002 (3)	0.0624 (10)	
C6A	0.15779 (17)	0.4555 (4)	0.8857 (4)	0.0653 (11)	
C7A	0.19146 (19)	0.3778 (5)	0.9810 (4)	0.0726 (11)	
C8A	0.1903 (2)	0.2153 (5)	0.9877 (4)	0.0789 (12)	
H8AA	0.2136	0.1628	1.0506	0.095*	
C9A	0.1552 (2)	0.1265 (5)	0.9028 (4)	0.0817 (13)	
H9AA	0.1546	0.0141	0.9089	0.098*	
C10A	0.12156 (19)	0.2003 (4)	0.8105 (4)	0.0738 (12)	
H10A	0.0974	0.1390	0.7538	0.089*	
C11A	0.2637 (2)	0.4050 (6)	1.1506 (4)	0.1000 (15)	
H11A	0.2848	0.4887	1.1984	0.150*	
H11B	0.2954	0.3379	1.1142	0.150*	
H11C	0.2380	0.3403	1.2028	0.150*	
O1B	0.43692 (11)	0.4470 (4)	0.56095 (19)	0.0886 (11)	
O2B	0.38305 (12)	0.3658 (4)	0.7646 (2)	0.0954 (11)	
N1B	0.43025 (14)	0.5949 (5)	0.3500 (3)	0.0882 (13)	
C1B	0.4797 (2)	0.5579 (8)	0.0265 (4)	0.114 (2)	
H1B1	0.4393	0.5657	−0.0206	0.137*	0.659 (8)
H1B2	0.5001	0.6636	0.0282	0.137*	0.659 (8)
C2B	0.4685 (3)	0.5001 (9)	0.1557 (4)	0.099 (2)	0.659 (8)
H2B1	0.4326	0.4251	0.1547	0.119*	0.659 (8)
H2B2	0.5063	0.4440	0.1885	0.119*	0.659 (8)
C3B	0.4545 (2)	0.6411 (7)	0.2340 (3)	0.1071 (19)	
H3B1	0.4935	0.7037	0.2480	0.128*	0.659 (8)
H3B2	0.4233	0.7099	0.1911	0.128*	0.659 (8)
H1C1	0.4538	0.6014	−0.0417	0.137*	0.341 (8)
H1C2	0.5080	0.6450	0.0539	0.137*	0.341 (8)
C2C	0.4343 (5)	0.5349 (17)	0.1277 (7)	0.099 (2)	0.341 (8)
H2C1	0.4345	0.4224	0.1534	0.119*	0.341 (8)
H2C2	0.3910	0.5628	0.0989	0.119*	0.341 (8)
H3C1	0.5011	0.6418	0.2414	0.128*	0.341 (8)
H3C2	0.4406	0.7509	0.2158	0.128*	0.341 (8)
C4B	0.37526 (17)	0.6398 (6)	0.3855 (4)	0.0861 (14)	
H4BA	0.3505	0.7030	0.3311	0.103*	
C5B	0.34914 (16)	0.6039 (6)	0.4962 (4)	0.0798 (13)	
C6B	0.38292 (16)	0.5038 (6)	0.5797 (3)	0.0772 (12)	
C7B	0.35069 (17)	0.4685 (6)	0.6899 (4)	0.0813 (13)	
C8B	0.2942 (2)	0.5351 (6)	0.7126 (5)	0.0921 (15)	
H8BA	0.2747	0.5107	0.7857	0.110*	
C9B	0.2641 (2)	0.6386 (6)	0.6309 (6)	0.1033 (18)	
H9BA	0.2254	0.6868	0.6500	0.124*	
C10B	0.29012 (18)	0.6703 (6)	0.5244 (5)	0.0971 (15)	
H10B	0.2687	0.7376	0.4679	0.116*	
C11B	0.3548 (2)	0.3244 (8)	0.8761 (3)	0.1133 (19)	
H11D	0.3812	0.2460	0.9193	0.170*	
H11E	0.3129	0.2795	0.8589	0.170*	
H11F	0.3511	0.4196	0.9259	0.170*	
H1O	0.131 (2)	0.652 (5)	0.808 (4)	0.103 (16)*	
H2O	0.455 (2)	0.543 (5)	0.415 (4)	0.110 (15)*	

### Atomic displacement parameters (Å^2^)

**Table:**

	*U*^11^	*U*^22^	*U*^33^	*U*^12^	*U*^13^	*U*^23^
O1A	0.0662 (16)	0.0594 (16)	0.0884 (19)	−0.0094 (12)	0.0296 (15)	−0.0089 (13)
O2A	0.088 (2)	0.092 (2)	0.090 (2)	−0.0109 (17)	0.0239 (17)	−0.0061 (17)
N1A	0.0681 (19)	0.0525 (19)	0.080 (2)	0.0005 (15)	0.0308 (17)	−0.0061 (15)
C1A	0.071 (2)	0.0507 (19)	0.075 (2)	−0.0066 (17)	0.040 (2)	−0.0103 (17)
C2A	0.070 (2)	0.046 (2)	0.083 (3)	−0.0049 (17)	0.036 (2)	−0.0073 (18)
C3A	0.087 (3)	0.055 (2)	0.081 (3)	0.003 (2)	0.025 (2)	−0.011 (2)
C4A	0.069 (2)	0.053 (2)	0.075 (3)	−0.0054 (18)	0.042 (2)	−0.0106 (18)
C5A	0.066 (2)	0.054 (2)	0.069 (2)	−0.0058 (18)	0.0380 (19)	−0.0024 (19)
C6A	0.063 (2)	0.054 (2)	0.082 (3)	−0.0068 (18)	0.042 (2)	−0.005 (2)
C7A	0.069 (2)	0.074 (3)	0.078 (3)	−0.006 (2)	0.041 (2)	0.000 (2)
C8A	0.080 (3)	0.074 (3)	0.086 (3)	0.003 (2)	0.044 (2)	0.009 (2)
C9A	0.101 (3)	0.061 (2)	0.087 (3)	−0.002 (2)	0.052 (3)	0.011 (2)
C10A	0.086 (3)	0.056 (2)	0.083 (3)	−0.010 (2)	0.047 (2)	−0.008 (2)
C11A	0.099 (3)	0.130 (4)	0.073 (3)	0.002 (3)	0.025 (3)	0.015 (3)
O1B	0.0384 (13)	0.189 (3)	0.0393 (13)	0.0166 (16)	0.0074 (10)	0.0170 (16)
O2B	0.0551 (15)	0.188 (3)	0.0445 (14)	−0.0037 (18)	0.0181 (12)	0.0093 (17)
N1B	0.0439 (18)	0.179 (4)	0.0413 (17)	0.000 (2)	−0.0026 (13)	0.026 (2)
C1B	0.060 (3)	0.224 (6)	0.060 (3)	0.009 (3)	0.021 (2)	0.022 (3)
C2B	0.041 (4)	0.201 (7)	0.057 (3)	0.025 (4)	0.016 (3)	0.030 (4)
C3B	0.067 (3)	0.215 (6)	0.038 (2)	−0.013 (3)	−0.0081 (18)	0.040 (3)
C1C	0.060 (3)	0.224 (6)	0.060 (3)	0.009 (3)	0.021 (2)	0.022 (3)
C2C	0.041 (4)	0.201 (7)	0.057 (3)	0.025 (4)	0.016 (3)	0.030 (4)
C3C	0.067 (3)	0.215 (6)	0.038 (2)	−0.013 (3)	−0.0081 (18)	0.040 (3)
C4B	0.041 (2)	0.139 (4)	0.077 (3)	−0.009 (2)	−0.0144 (19)	0.023 (3)
C5B	0.0350 (18)	0.129 (4)	0.076 (3)	−0.004 (2)	0.0069 (18)	0.014 (2)
C6B	0.0396 (19)	0.140 (4)	0.053 (2)	0.004 (2)	0.0116 (16)	0.001 (2)
C7B	0.045 (2)	0.139 (4)	0.060 (2)	−0.009 (2)	0.0191 (18)	−0.005 (2)
C8B	0.065 (3)	0.108 (3)	0.107 (4)	−0.014 (3)	0.046 (3)	−0.012 (3)
C9B	0.053 (2)	0.097 (3)	0.164 (5)	−0.011 (2)	0.054 (3)	−0.005 (3)
C10B	0.047 (2)	0.101 (3)	0.143 (4)	−0.005 (2)	0.018 (3)	0.015 (3)
C11B	0.086 (3)	0.205 (6)	0.051 (2)	−0.024 (3)	0.032 (2)	0.007 (3)

### Geometric parameters (Å, °)

**Table:**

O1A—C6A	1.360 (4)	O2B—C7B	1.366 (5)
O1A—H1O	1.05 (5)	O2B—C11B	1.443 (4)
O2A—C7A	1.353 (5)	N1B—C4B	1.306 (5)
O2A—C11A	1.446 (5)	N1B—C3B	1.462 (5)
N1A—C4A	1.283 (4)	N1B—H2O	0.98 (5)
N1A—C3A	1.459 (5)	C1B—C1B^ii^	1.443 (11)
C1A—C2A	1.508 (5)	C1B—C2B	1.545 (6)
C1A—C1A^i^	1.531 (7)	C1B—H1B1	0.9900
C1A—H1A1	0.9900	C1B—H1B2	0.9900
C1A—H1A2	0.9900	C2B—C3B	1.510 (6)
C2A—C3A	1.513 (5)	C2B—H2B1	0.9900
C2A—H2AA	0.9900	C2B—H2B2	0.9900
C2A—H2AB	0.9900	C3B—H3B1	0.9900
C3A—H3AA	0.9900	C3B—H3B2	0.9900
C3A—H3AB	0.9900	C2C—H2C1	0.9900
C4A—C5A	1.447 (5)	C2C—H2C2	0.9900
C4A—H4AA	0.9500	C4B—C5B	1.404 (6)
C5A—C6A	1.398 (5)	C4B—H4BA	0.9500
C5A—C10A	1.411 (5)	C5B—C10B	1.421 (6)
C6A—C7A	1.415 (6)	C5B—C6B	1.427 (6)
C7A—C8A	1.372 (6)	C6B—C7B	1.459 (5)
C8A—C9A	1.397 (6)	C7B—C8B	1.359 (6)
C8A—H8AA	0.9500	C8B—C9B	1.396 (7)
C9A—C10A	1.376 (6)	C8B—H8BA	0.9500
C9A—H9AA	0.9500	C9B—C10B	1.354 (7)
C10A—H10A	0.9500	C9B—H9BA	0.9500
C11A—H11A	0.9800	C10B—H10B	0.9500
C11A—H11B	0.9800	C11B—H11D	0.9800
C11A—H11C	0.9800	C11B—H11E	0.9800
O1B—C6B	1.269 (4)	C11B—H11F	0.9800
			
C6A—O1A—H1O	107 (3)	C4B—N1B—C3B	122.9 (4)
C7A—O2A—C11A	117.6 (4)	C4B—N1B—H2O	111 (3)
C4A—N1A—C3A	118.4 (3)	C3B—N1B—H2O	125 (3)
C2A—C1A—C1A^i^	114.3 (4)	C1B^ii^—C1B—C2B	106.5 (6)
C2A—C1A—H1A1	108.7	C1B^ii^—C1B—H1B1	110.4
C1A^i^—C1A—H1A1	108.7	C2B—C1B—H1B1	110.4
C2A—C1A—H1A2	108.7	C1B^ii^—C1B—H1B2	110.4
C1A^i^—C1A—H1A2	108.7	C2B—C1B—H1B2	110.4
H1A1—C1A—H1A2	107.6	H1B1—C1B—H1B2	108.6
C1A—C2A—C3A	112.1 (3)	C3B—C2B—C1B	109.3 (5)
C1A—C2A—H2AA	109.2	C3B—C2B—H2B1	109.8
C3A—C2A—H2AA	109.2	C1B—C2B—H2B1	109.8
C1A—C2A—H2AB	109.2	C3B—C2B—H2B2	109.8
C3A—C2A—H2AB	109.2	C1B—C2B—H2B2	109.8
H2AA—C2A—H2AB	107.9	H2B1—C2B—H2B2	108.3
N1A—C3A—C2A	112.2 (3)	N1B—C3B—C2B	112.6 (5)
N1A—C3A—H3AA	109.2	N1B—C3B—H3B1	109.1
C2A—C3A—H3AA	109.2	C2B—C3B—H3B1	109.1
N1A—C3A—H3AB	109.2	N1B—C3B—H3B2	109.1
C2A—C3A—H3AB	109.2	C2B—C3B—H3B2	109.1
H3AA—C3A—H3AB	107.9	H3B1—C3B—H3B2	107.8
N1A—C4A—C5A	122.5 (4)	H2C1—C2C—H2C2	108.3
N1A—C4A—H4AA	118.8	N1B—C4B—C5B	126.5 (4)
C5A—C4A—H4AA	118.8	N1B—C4B—H4BA	116.8
C6A—C5A—C10A	119.2 (4)	C5B—C4B—H4BA	116.8
C6A—C5A—C4A	120.4 (3)	C4B—C5B—C10B	119.6 (4)
C10A—C5A—C4A	120.4 (4)	C4B—C5B—C6B	119.4 (3)
O1A—C6A—C5A	121.8 (4)	C10B—C5B—C6B	121.0 (4)
O1A—C6A—C7A	118.2 (4)	O1B—C6B—C5B	123.4 (3)
C5A—C6A—C7A	120.1 (3)	O1B—C6B—C7B	121.4 (4)
O2A—C7A—C8A	126.0 (5)	C5B—C6B—C7B	115.2 (3)
O2A—C7A—C6A	114.4 (4)	C8B—C7B—O2B	125.2 (4)
C8A—C7A—C6A	119.6 (4)	C8B—C7B—C6B	121.4 (4)
C7A—C8A—C9A	120.5 (4)	O2B—C7B—C6B	113.4 (3)
C7A—C8A—H8AA	119.7	C7B—C8B—C9B	121.5 (4)
C9A—C8A—H8AA	119.7	C7B—C8B—H8BA	119.2
C10A—C9A—C8A	120.6 (4)	C9B—C8B—H8BA	119.2
C10A—C9A—H9AA	119.7	C10B—C9B—C8B	120.0 (4)
C8A—C9A—H9AA	119.7	C10B—C9B—H9BA	120.0
C9A—C10A—C5A	120.0 (4)	C8B—C9B—H9BA	120.0
C9A—C10A—H10A	120.0	C9B—C10B—C5B	120.8 (5)
C5A—C10A—H10A	120.0	C9B—C10B—H10B	119.6
O2A—C11A—H11A	109.5	C5B—C10B—H10B	119.6
O2A—C11A—H11B	109.5	O2B—C11B—H11D	109.5
H11A—C11A—H11B	109.5	O2B—C11B—H11E	109.5
O2A—C11A—H11C	109.5	H11D—C11B—H11E	109.5
H11A—C11A—H11C	109.5	O2B—C11B—H11F	109.5
H11B—C11A—H11C	109.5	H11D—C11B—H11F	109.5
C7B—O2B—C11B	117.3 (3)	H11E—C11B—H11F	109.5

Symmetry codes: (i) −*x*, −*y*+2, −*z*+1; (ii) −*x*+1, −*y*+1, −*z*.

### Hydrogen-bond geometry (Å, °)

**Table:**

*D*—H···*A*	*D*—H	H···*A*	*D*···*A*	*D*—H···*A*
O1A—H1O···N1A	1.05 (5)	1.64 (5)	2.575 (4)	146 (4)
N1B—H2O···O1B	0.98 (5)	1.87 (5)	2.655 (4)	136 (4)
N1B—H2O···O1B^iii^	0.98 (5)	2.31 (5)	2.976 (4)	125 (4)

Symmetry codes: (iii) −*x*+1, −*y*+1, −*z*+1.
